# The UCSC Genome Browser database: 2024 update

**DOI:** 10.1093/nar/gkad987

**Published:** 2023-11-11

**Authors:** Brian J Raney, Galt P Barber, Anna Benet-Pagès, Jonathan Casper, Hiram Clawson, Melissa S Cline, Mark Diekhans, Clayton Fischer, Jairo Navarro Gonzalez, Glenn Hickey, Angie S Hinrichs, Robert M Kuhn, Brian T Lee, Christopher M Lee, Phillipe Le Mercier, Karen H Miga, Luis R Nassar, Parisa Nejad, Benedict Paten, Gerardo Perez, Daniel Schmelter, Matthew L Speir, Brittney D Wick, Ann S Zweig, David Haussler, W James Kent, Maximilian Haeussler

**Affiliations:** Genomics Institute, University of California Santa Cruz, Santa Cruz, CA 95064, USA; Genomics Institute, University of California Santa Cruz, Santa Cruz, CA 95064, USA; Institute of Neurogenomics, Helmholtz Zentrum München GmbH - German Research Center for Environmental Health, 85764 Neuherberg, Germany; Medical Genetics Center (Medizinisch Genetisches Zentrum), Munich 80335, Germany; Genomics Institute, University of California Santa Cruz, Santa Cruz, CA 95064, USA; Genomics Institute, University of California Santa Cruz, Santa Cruz, CA 95064, USA; Genomics Institute, University of California Santa Cruz, Santa Cruz, CA 95064, USA; Genomics Institute, University of California Santa Cruz, Santa Cruz, CA 95064, USA; Genomics Institute, University of California Santa Cruz, Santa Cruz, CA 95064, USA; Genomics Institute, University of California Santa Cruz, Santa Cruz, CA 95064, USA; Genomics Institute, University of California Santa Cruz, Santa Cruz, CA 95064, USA; Genomics Institute, University of California Santa Cruz, Santa Cruz, CA 95064, USA; Robert Kuhn Consulting, Aptos,CA 95003, USA; Genomics Institute, University of California Santa Cruz, Santa Cruz, CA 95064, USA; Genomics Institute, University of California Santa Cruz, Santa Cruz, CA 95064, USA; Swiss-Prot Group, SIB Swiss Institute of Bioinformatics, CMU, 1 Michel Servet, 1211 Geneva 4, Switzerland; Genomics Institute, University of California Santa Cruz, Santa Cruz, CA 95064, USA; Genomics Institute, University of California Santa Cruz, Santa Cruz, CA 95064, USA; Genomics Institute, University of California Santa Cruz, Santa Cruz, CA 95064, USA; Genomics Institute, University of California Santa Cruz, Santa Cruz, CA 95064, USA; Genomics Institute, University of California Santa Cruz, Santa Cruz, CA 95064, USA; Genomics Institute, University of California Santa Cruz, Santa Cruz, CA 95064, USA; Genomics Institute, University of California Santa Cruz, Santa Cruz, CA 95064, USA; Genomics Institute, University of California Santa Cruz, Santa Cruz, CA 95064, USA; Genomics Institute, University of California Santa Cruz, Santa Cruz, CA 95064, USA; Genomics Institute, University of California Santa Cruz, Santa Cruz, CA 95064, USA; Genomics Institute, University of California Santa Cruz, Santa Cruz, CA 95064, USA; Genomics Institute, University of California Santa Cruz, Santa Cruz, CA 95064, USA

## Abstract

The UCSC Genome Browser (https://genome.ucsc.edu) is a web-based genomic visualization and analysis tool that serves data to over 7,000 distinct users per day worldwide. It provides annotation data on thousands of genome assemblies, ranging from human to SARS-CoV2. This year, we have introduced new data from the Human Pangenome Reference Consortium and on viral genomes including SARS-CoV2. We have added 1,200 new genomes to our GenArk genome system, increasing the overall diversity of our genomic representation. We have added support for nine new user-contributed track hubs to our public hub system. Additionally, we have released 29 new tracks on the human genome and 11 new tracks on the mouse genome. Collectively, these new features expand both the breadth and depth of the genomic knowledge that we share publicly with users worldwide.

## Graphical Abstract

**Graphical Abstract:**
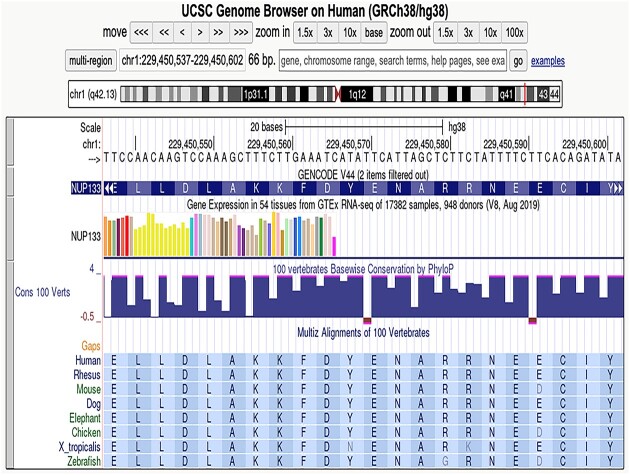

## Introduction

When the UCSC Genome Browser (GB) was first introduced in 2000, its support was limited to one draft human assembly. In the intervening 23 years, we have added 222 additional mostly vertebrate assemblies. Meanwhile, we have greatly expanded the functionality of the genome browser and its related tools to support genomic analysis by our users. While other genome browsers are available (1–4), the UCSC Genome Browser is one of the most widely-used, with over 7,000 unique users a day.

Currently the GB serves over 65,000 tracks of annotation data from labs worldwide. While the majority of these tracks (37,723) are on human genome assemblies, we also have 9,760 tracks on mouse genome assemblies and 19,664 tracks on other genomes.

The UCSC Genome Browser maintains an active email support list. We receive over 700 questions a year, each of which we answer quickly and carefully, usually under three workdays. For ways to contact us, see https://genome.ucsc.edu/contacts.html

While many of our users work strictly with annotations that are provided by the browser, a significant portion of our user base also works with custom annotations provided as track hubs (5) or custom tracks. Our users have created or accessed 62,000 track hubs over the past year. We currently host over 15,000 custom tracks with over 50 Gb of user data. Further, we currently list 113 public track hubs that have been contributed by our users and provide access to a wide variety of data.

Another popular feature of the GB is the ability to transfer annotations from one genome to another. Our liftOver technology makes it easy to map annotations between genomes. This technology is enabled by pairwise alignments of hundreds of assemblies. See https://genome.ucsc.edu/cgi-bin/hgLiftOver for further details.

Our API provides programmatic access to all of our tables and public hubs, and is accessed by roughly 500 users per day. See https://genome.ucsc.edu/goldenPath/help/api.html for further details.

Our saved sessions technology enables users to save configurations for future access, or to share browser views with colleagues and to provide interactive access to data in publications. We currently maintain more than 200,000 saved sessions, 40,000 of which have been accessed in the last year. See https://genome.ucsc.edu/goldenPath/help/hgSessionHelp.html for further details..

In addition to the UCSC Genome Browser itself, we provide a wide variety of command line tools for genomic analysis. This year, we introduced a GitHub repository which provides open source access to these tools, under the terms of the MIT open source software license. See https://github.com/ucscGenomeBrowser/kent-core for the repository, and https://hgdownload.soe.ucsc.edu/downloads.html#utilities_downloads for further information on these tools.

## New and updated annotations

This year, we have introduced new annotations including tracks to share data from the Human Pangenome Reference Consortium, new gene tracks, tracks to aid in sequencing projects and to help identify sequencing errors.

### Pangenome tracks

The Human Pangenome Reference Consortium has produced 47 high-quality diploid genome assemblies from a genetically diverse set of individuals (6). This year, we have unveiled four new tracks that summarize these data.

**Figure 1.**
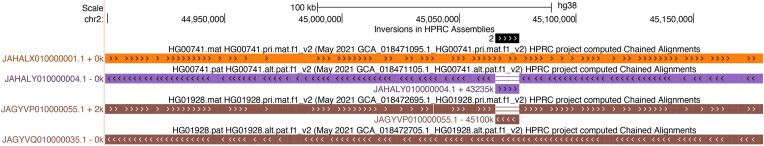
Browser view of a section of hg38 chromosome 2 where the HPRC Inversions summary track shows that there are two inversions in the HPRC genomes. Also shown are four chain tracks for both chromosomes of two individuals (HG00741, HG01928) showing that each individual has one parent with the inversion, whereas the other parent doesn’t.

To summarize the base-level differences in the 90 sequences used to build the Pangenome variation graph (this excludes the sequences of two individuals, which were left out by HPRC to verify the process) we have the HPRC VCF track. To summarize the inversions (Figure 1) and local duplications with respect to hg38 we have the HPRC Rearrangements track on these two assemblies. To visualize the multiple alignment that gives rise to the HPRC VCF track we have the HPRC MAF track. To visualize the pairwise alignments that were extracted from the larger human pangenome variation graph, we have the HPRC chains track.

### Other new annotations

#### Gene sets

Our two most widely-used gene tracks on human (hg38) and mouse (mm39) were built with our knownGene pipeline applied to the Ensembl/GENCODE transcripts. These are now the default gene tracks for those assemblies. Since the knownGene pipeline builds extensive associations from the annotations, we can now share additional metadata for each item as well as link to external resources.

To increase the gene annotation on assemblies that are otherwise poorly annotated, we now provide TOGA (Tool to infer Orthologs from Genome Alignments) gene prediction tracks for 41 of our full assemblies and more than 600 GenArk assemblies (see below). TOGA is a homology-based method that integrates gene annotation, inferring orthologs and classifying genes as *intact* or *lost*. TOGA was produced by Michael Hiller and colleagues (7). Its open-source software is available at github.com/hillerlab/TOGA.

The **HGNC** track on hg38 is a Genes track from the HUGO Gene Nomenclature Committee, the internationally recognized body for standardizing gene symbols and names (8). This searchable track is a unifier of different identifiers for the same gene, with up to 24 aliases linked together under one annotation. Hovering over a gene in this track shows all symbols and aliases. Clicking into an entry will provide information on the current names, former names and full names. We have added this thesaurus archive to our search engine to help researchers find genes under any of these aliases.

#### Sequencing aids

To aid in the interpretation of sequencing results, we introduce the Problematic Regions composite track. This track shows regions which are problematic or special cases for sequencing, as well as highly variable regions for GRCh38/hg38. Its four subtracks include the **ENCODE Blacklist** by Anshul Kundaje (9) and the GRC (Genome Reference Consortium) Exclusions (http://genomeref.blogspot.com/2021/07/one-of-these-things-doest-belong.html). The UCSC Unusual Regions track annotates the well-known gene clusters such as T-Cell Receptors, protocadherins, HOX, MHC, immunoglobulins or regions with special properties such as the PAR region. The Highly Reproducible Regions (10) composite track highlights regions and variants from eight samples that can be used to assess variant calls and variant detection pipelines.

#### Variation

This year, we have introduced the Recombination Rate supertrack. This supertrack represents calculated rates of recombination based on the genetic maps from deCODE and 1000 Genomes. It includes three subtracks with the deCODE recombination rates (paternal, maternal and average); and one subtrack with the 1,000 Genomes recombination rate, which was lifted from hg19 and can be used as a drop-in replacement for the GRCh37/hg19 track. Note that the deCODE recombination rate data is newer and has a higher resolution. The Recombination Rate supertrack also contains two more subtracks from deCODE: one with the raw data of all cross-overs tagged with their proband ID and one with around 8000 human *de novo* mutation variants that are linked to cross-over changes.

The Genome Aggregation Database (gnomAD) Constraint Metrics track (11) identifies genomic locations where mutations are likely to be deleterious. It shows metrics of pathogenicity per-gene as predicted for gnomAD v2.1.1 and identifies genes subject to strong selection against various classes of mutation. Previously, this track was available only on hg19. Now, it is also available on GRCh38/hg38.

The Constraint score container supertrack now contains the UK Biobank Depletion rank score track (12) for GRCh38/hg38. This track is part of the Constraint score container track that includes several subtracks showing the results of various constraint prediction algorithms.

For assessing common nucleotide variation in human, we now offer NCBI’s **dbSNP build 155** (13) for the GRCh38/hg38 and GRCh37/hg19 human assemblies. This dbSNP release reaches a new milestone of over 1 billion RefSNP (rs) records. For hg38 (GRCh38), approximately 998 million distinct variants (RefSNP clusters with rs# ids) have been mapped to more than 1.06 billion genomic locations including alternate haplotype and fix patch sequences. Further, dbSNP remapped variants from hg38 to hg19 (GRCh37); in total, approximately 981 million distinct variants were mapped to more than 1.02 billion genomic locations including alternate haplotype and fix patch sequences.

To identify structural variants within healthy humans we offer the DGV Gold Standard track for hg38. This track displays curated variants from a selected number of studies in the Database of Genomic Variants (DGV) with a criterion that requires a variant to be found in at least two different studies and found in at least two different samples.

To aid genomics research on non-human animals we released the EVA SNP release 4 tracks for 23 non-human assemblies. These tracks contain mappings of single nucleotide variants and small insertions and deletions (indels) from the European Variation Archive (EVA) Release 4 (14).

#### Expression

To help researchers get a comprehensive overview of gene expression across tissue types we released as native tracks the FANTOM5 promoter level expression data (15) for hg19, hg38, mm10, canFam3, rheMac8, rn6 and galGal5. The FANTOM5 tracks show mapped transcription start sites (TSS) and their usage in primary cells, cell lines and tissues. These tracks were created as a track hub by the FANTOM5 project itself and we copied their track hub to increase stability and performance.

On the human assemblies, the annotation tracks for JASPAR (16) and ReMap (17) also were copied from these resources rather than created by us. We hope that track hubs will be adopted by more projects in the future and will simplify the exchange and curation of genome annotations.

To give our users access to human single cell RNA-Seq on human tissues from the Genotype-Tissue Expression (GTEx) project we released the Single-Nuclei Cross-Tissue Map (18) supertrack for the human assembly GRCh38/hg38. This track collection contains three bar chart tracks of RNA expression. The first track, **Cross Tissue Nucle**i, allows cells to be grouped together and faceted on up to 4 categories: tissue, cell class, cell subclass and cell type. The second track, **Cross Tissue Details**, allows cells to be grouped together and faceted on up to 7 categories: tissue, cell class, cell subclass, cell type, granular cell type, sex and donor. The third track, **GTEx Immune Atlas**, allows cells to be grouped together and faceted on up to 5 categories: tissue, cell type, cell class, sex and donor.

To support phenotypic interpretation, we’ve added the **PanelApp track** from Genomics England (19). This track shows expert, crowdsourced diagnostic disease panels among genes, copy-number variants (CNV) and short tandem repeats (STR). This collection of nearly 50,000 associations includes a confidence level color-score, detailed mouseover, inheritance patterns and links to the primary data source.

#### Comparative genomics

We added a new multiple alignment with 470 mammal assemblies to GRCh38/hg38. This composite track displays multiple alignments (Multiz) and measurements of evolutionary conservation (phastCons and phyloP) for 470 mammals. It is the first major whole-genome alignment that was produced with our lastz/chain/net/multiz pipeline externally, by the group of Michael Hiller.

## New assemblies and patches

This year we released new curated assembly hubs for human (T2T-CHM13/hs1) and for the Mpox virus (MT903340.1/mpxvRivers). These two assemblies showcase our new approaches for releasing assemblies on the UCSC Genome Browser by hosting them in track hubs rather than a mySQL database. Curated assemblies on track hubs will be reviewed by our quality assurance team in the same manner as previous mySQL assemblies, but use our track hub technology. Most users will perceive no difference between the curated hubs and mySQL assemblies. However, by leveraging the track hub technology, the curated track hub approach offers greater scalability, which will ultimately translate to support for more assemblies.

We also released GRCh38 patch release 14 to the hg38 assembly. hg38 has been updated with patches since its release in 2013. The GRC patch releases do not change any previously existing sequences but add new sequences for ‘fix patches’ or alternate haplotypes in specific regions of the main chromosome sequences. While these patches introduce more duplication, which may complicate some analyses, they are unlikely to make a difference for most users, yet they offer a more comprehensive representation of the human genome.

### Viral genome data

Beginning with the SARS virus in 2015, whenever we perceive a need in the community for genomic resources on an emergent disease, we expedite production of a genome browser. We have produced browsers for the Ebola virus and for SARS COV-2.This year, we launched the **MPox browser**, which contains several tracks useful for virologists, including Genbank alignments, a Transcriptome stage track and sequencing primers.

This year we helped develop a recombination rate track for the **SARS-CoV-2** browser based on a new method for detecting recombination in pandemic-scale phylogenies (20).

### GenArk

Recently we introduced the Genome Archive (GenArk) collection (21) of UCSC Genome Browsers for assemblies hosted at NCBI (22). Each of these assemblies comes with BLAT (23) support. Since then we have been adding new assemblies to our GenArk Assembly system by the hundreds every year. We currently have over three thousand assemblies in the system. This year we added the genomic assemblies of the Human Pangenome Reference Consortium, the California Conservation Genomics Project (24) assemblies and the Vertebrate Genomes Project (25) assemblies. We also added all 264 reference viral genomes from NCBI that are human pathogens (https://hgdownload.soe.ucsc.edu/hubs/viral/index.html), a selection made possible thanks to a collaboration with the curators of Viralzone.org.

### New displays

#### SquishyPack

**Figure 2.**
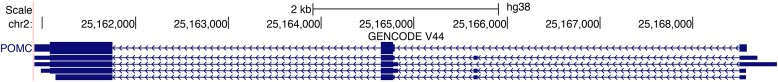
Illustration of the squishyPack display mode on the GENCODE gene set.

One of our fundamental challenges is devising approaches to display data in ways that are informative yet demand less screen space. One common user concern is that our gene tracks display far too many transcripts. To address this, we have created a new track display mode called *squishyPack*, designed especially for transcript data. In this mode, the first item (transcript) is shown at full height and with labels, while others are shown without labels and at half-height (Figure 2). We now utilize this mode in our default GENCODE gene track, leveraging GENCODE’s transcript rank to select the top-ranked transcript This functionality is available to all bigBed-based tracks, with the track developer indicating the track item priorities through settings in the *trackDb* track configuration file.

See https://genome.ucsc.edu/goldenPath/help/bigBed.html#Ex4 for further information.

#### Sequence logos

**Figure 3.**
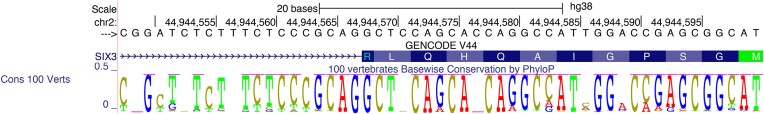
Sequence logos summarizing the genomic conservation at each genomic position. The height of each letter at each position describes the conservation at that position and the observed nucleotides.

This year we have introduced the ability for tracks to display sequence logos. These logos are either created dynamically from wiggle and multiZ tracks or are specified in advance with four wiggle tracks specifying the height of the A, C, G and T nucleotides in each genomic position. We leverage this functionality with dynamically-created sequence logos in the phastOdds and phastCons tracks of the multiz100way and multiz30way conservation tracks on hg38 (Figure 3).

See https://genome.ucsc.edu/goldenPath/help/bigWig.html#dynseq for more information.

#### Rearrangement mode in BLAT results and chains

**Figure 4.**
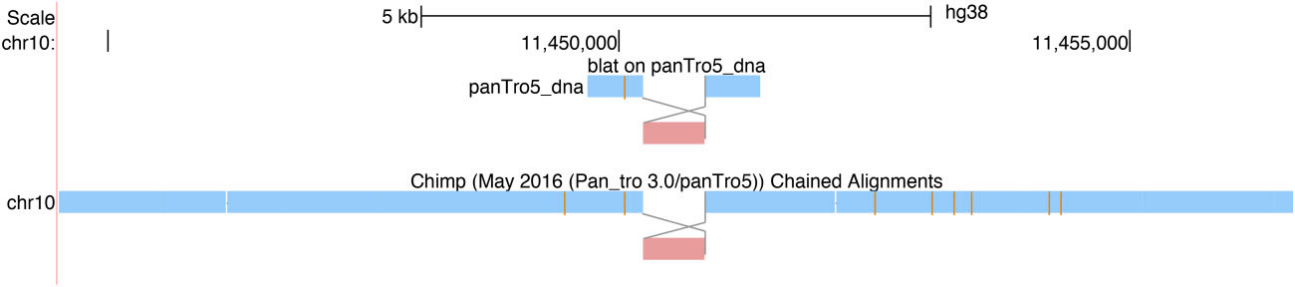
Illustration of Rearrangement Mode, displaying a region on hg38 chromosome 10 where a block is inverted in the pairwise alignment of a BLAT query sequence and in an alignment to Chimps chromosome 10.

One challenge in analyzing rearrangements in chain and PSL tracks is that one cannot see the order of homologies in the query sequence. To address this, we now provide the option of *rearrangement mode* or *snake mode* in chain and PSL/BLAT displays (Figure 4). In this mode, one can follow lines between aligned blocks in two sequences to see the relative orientation of the blocks. To turn it on select ‘rearrangement mode’ as described at https://genome.ucsc.edu/goldenPath/help/chain.html#rearrangement.

#### Decorators

**Figure 5.**
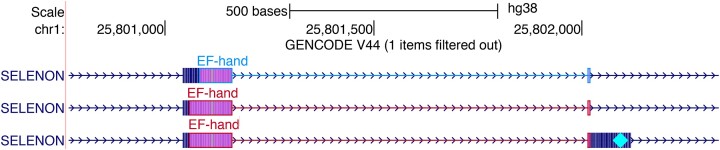
Decorator visualization of EF-Hand domains in the gene SELENON. The upper transcript annotation, in blue, was generated by TrEMBL, while the lower two transcripts in red are annotated by Swiss-Prot. A cyan diamond in the right exon marks the location of a selenocysteine.

Decorators provide a way to augment a linkedFeatures track by highlighting regions of individual transcripts with designated glyphs (Figure 5). LinkedFeatures tracks include BED, bigBed, PSL and bigGenePred and Decorator functionality is available for any of these tracks. The decorations are specified using additional fields in bigBed files. See https://genome.ucsc.edu/goldenPath/help/decorator.html for further information.

## Training and contact information

### Tutorial

We have introduced an interactive tutorial for new users. The tutorial covers topics including navigation, configuring track display settings, searching for tracks and viewing the negative strand (3′ to 5′). This interactive tutorial is available via the ‘Help’ drop-down menu in the toolbar.

### Teaching module

In response to requests from Browser users, we developed a new teaching module designed to assist teachers and students in understanding selected topics in Molecular Biology, Genetics, Medicine, Population Biology and Evolution. Using the Genome Browser as a display device, the new module is organized as short stories written by undergraduates for undergraduates. Supported by numerous links to live Browser sessions, the stories tell the tales and show some of the capabilities of the Genome Browser.

See https://genome.ucsc.edu/training/education/.

### Email support

We are very proud of our quick and complete responses to questions emailed to us by our users at genome@ucsc.edu, and we encourage all users to reach out to us as questions arise.

## Future plans

### Native disk hosting

As fewer universities are currently providing web hosting services to their researchers, we are frequently asked for our advice on where our users can host their track hubs. In 2024, we will implement a system wherein our users can directly store their track hubs on our systems. As well as providing users with a convenient storage solution, this will reduce the data transfer latency and the time required for rendering these users’ data

### Automatic lifting of annotations

In the very near future there will be thousands of human genomes in the public domain plus tens of thousands of additional genomes which will not be available publicly. To annotate these genomes efficiently, while leveraging the breadth of annotation data available on hg38, we will implement a method of lifting annotations to unannotated genomes ‘on-the-fly’ without the need to ‘manually’ lift over tracks.

## Data Availability

The UCSC Genome Browser (https://genome.ucsc.edu/) is freely available to all users. The source code for the Genome Browser, Blat utility, liftOver utility and other utilities which are free for non-profit academic research and for personal use, and available to commercial users by license.

## Notes

Present address: Brian Raney, Genomics Institute, University of California Santa Cruz, 1156 High Street, Santa Cruz, CA 95064, USA.
